# Mixed-methods study to assess an intervention for healthier lifestyles in primary healthcare

**DOI:** 10.1093/eurpub/ckac129.325

**Published:** 2022-10-25

**Authors:** A Bytyci Katanolli, S Merten, M Kwiatkowski, K Obas, J Gerold, M Zahorka, N Jerliu, Q Ramadani, N Fota

**Affiliations:** 1 Epidemiology and Public Health, Swiss Tropical and Public Health Institute, Basel, Switzerland; 2 University of Basel, Medical Faculty, Basel, Switzerland; 3 Swiss Centre for International Health, Swiss Tropical and Public Health Institute, Basel, Switzerland; 4 National Institute of Public Health, Kosovo, Prishtina, Kosovo; 5 University of Prishtina, Medical Faculty, Prishtina, Kosovo; 6 Accessible Quality Healthcare Project, Prishtina, Kosovo

## Abstract

**Background:**

Smoking, physical inactivity, low fruit and vegetable consumption, and obesity are common in Kosovo. The Accessible Quality Healthcare project is implementing a primary healthcare intervention that entails nurse-guided motivational counselling to facilitate lifestyle behaviour change. This study assesses the uptake of counselling and the impact on health behaviours and participants’ stages of health behaviour change as well as describes experiences and perceived benefits of motivational counselling.

**Methods:**

Study participants (n = 907) were recruited from 12 municipalities participating in the Kosovo Non-Communicable Disease Cohort study. For the quantitative study, data on lifestyle behaviours, use of counselling, and stages for behavioural change were used. For the qualitative study, in-depth interviews were conducted with 26 cohort participants who had undergone motivational counselling.

**Results:**

Motivational counselling was received by only 22% of the eligible participants in the intervention municipalities. Unhealthy behaviours remain high even in persons who underwent counselling (of whom 13% are smokers; 86% are physically inactive; 93% with inadequate fruit and vegetable consumption; 61% are obese). According to the qualitative study results, the participants that received counselling were very satisfied with the services but requested additional services such as group physical activity sessions and specialized services for smoking cessation.

**Conclusions:**

More tailored and additional primary healthcare approaches in accordance with patients’ views need to be considered for the motivational counselling intervention to reach patients and efficiently facilitate lifestyle behaviour change.

**Key messages:**

• The following tailored approaches are suggested: a) strengthened referral mechanism b) specialized services for smoking cessation; c) delivery of group physical activity sessions.

• More tailored and additional primary health care approaches in accordance with patients’ views need to be considered for the motivational counselling intervention.

